# Mechanical Properties Study of Fe-Mn-Si Shape Memory Alloys Welding Seam Formed by Laser Welding with Filler Powder

**DOI:** 10.3390/ma11081454

**Published:** 2018-08-16

**Authors:** Heng Ju, Chengxin Lin, Yun Tian, Zhijie Liu, Huiling Jiang, Deping Sun

**Affiliations:** 1 Department of Mechanics, College of Naval Architecture & Ocean Engineering, Dalian Maritime University, No.1 Linghai Road, Dalian 116026, China; jh1990@dlmu.edu.cn (H.J.); liuzj2003@163.com (Z.L.); dlmu2017jhl@dlmu.edu.cn (H.J.); 2 Department of Technology, Beijing Satellite Manufacturer Limited Company, No. 18 Nansan Streer, Beijing 100094, China; tianyun20092010@163.com; 3 Department of Marine Engineering, College of Marine Engineering, Dalian Maritime University, No. 1 Linghai Road, Dalian 116026, China; sdpdmu@163.com

**Keywords:** laser welding, filler powder, Fe-Mn-Si SMAs, mechanical properties, strengthening mechanism

## Abstract

To reduce the residual stress and improve the fatigue property of the laser weldment by using the stress self-accommodation characteristic of Fe-Mn-Si shape memory alloys (SMAs), a Fe15Mn5Si12Cr6Ni memory alloy welding seam was formed inside 304 stainless steel by laser welding with filler powder. The combination of the hole-drilling method and the ANSYS software was used to research the distribution law of residual stress inside the laser welding specimen. The fatigue strength of the laser welded specimens with the Fe-Mn-Si SMAs welding seam (experimental materials) and 304 stainless steel welding seam (comparative materials) was measured by cycle bending fatigue test. The microhardness of the welding specimens was measured by the microhardness tester. The thermodynamic model of the laser welding process and the phase transition crystallography of Fe-Mn-Si SMAs were evaluated to analyze the strengthening mechanism of the mechanical properties in the experimental materials. The results show that the distribution law for residual stress in the experiment and simulation are consistent. The experimental materials possess low residual stress, high fatigue strength and high microhardness. The strengthening mechanism for mechanical properties is the welding residual stress-induced γ→ε martensitic transformation inside the experimental materials, which causes the tensile plastic strain of the welding seam to resist residual compression strain, and the residual stress, as the transition driving force, is released in shear processing.

## 1. Introduction

Laser welding has been rapidly developed and applied in recent years as a new welding method, and it possesses high energy density, fast heating and cooling speeds, a narrow welding seam and a small heat affected zone [1,2,3,4]. This technology not only effectively reduces intermetallic compounds inside the weldment, but also appropriately heats local micro-regions. Thus, laser welding is an ideal method for welding thin plates of the same or different kinds of materials; steels, titanium and aluminum, for example. However, while laser welding technology possesses many advantages for some materials, it also exhibits limitations in terms of processability. Due to the high thermal expansivity and carbon equivalent of the steel and the rapid heating and cooling speed of laser welding, high residual stress and element segregation occur, reducing the mechanical properties of the laser welding specimen. Because titanium alloy possesses low thermal expansivity and high laser absorptivity, it is easy to realize deep-fusion welding by laser welding titanium alloy. At high energy density, the metal will produce plasma by evaporation and ionization, reducing the welding stability. Furthermore, the titanium alloy will violently react with many active gases and form intermetallic compounds at high temperatures, which will decrease the comprehensive properties of the materials. Aluminum alloy has high thermal expansivity and laser reflectivity, and the process of laser welding aluminum alloy requires high laser power and an accurate beam focusing position; technologies have also been developed to improve the laser absorptivity of aluminum alloy. Furthermore, due to the low thermal conductivity of all of the materials and the concentrated thermal input of the laser, uneven expansion and contraction inside the weld metal and nearby metals will result in a variety of welding stresses and welding deformations. The combined effects of residual stresses on the welded joint and the external loads on the working medium will reduce the carrying capacity, fatigue strength and stress corrosion resistance of welded structures [5,6]. Therefore, it is necessary that the welding residual stress be measured and reduced to ensure the service security of the welded structure.

At present, the measuring methods for residual stress include the mechanical release method and the nondestructive method. The mechanical release method obtains the residual stress by measuring the stress variation in the process of cutting and separating the measuring part from the complete components. Although this method causes some damage inside the specimen, it possesses high measuring accuracy, a complete theory, and mature technology. Mechanical release methods are also widely used now, such as the hole-drilling method, ring-core method, sectioning method, and so on. These nondestructive methods do not damage the specimens, but have a high cost and require expensive equipment. The common methods include X-ray diffraction (XRD), neutron diffraction, ultrasonic method, magnetic method, and so on. In conclusion, the destructibility and expenditure of the hole-drilling method is small; thus, it is the most common method for residual stress measuring [7]. Considering the cost and the reliability of the residual stress measurement, a numerical simulation could analyze the temperature field and the stress field to verify the experimental results, and the ANSYS finite element software is widely applied. The ANSYS software is large-scale general finite element analysis software related to structure field, thermal field, electromagnetic field, flow field, coupled field, and so on. The software includes a pre-processing module, an analysis module, and a post-processing module, and the simulation results are obtained by the following procedures: establishment of model, definition of material property, setting of the initial conditions and boundary conditions, application of the load, solution, and so on.

The treatment methods to reduce the welding residual stress include post-weld heat treatment, vibration aging, and so on [8,9]. Although these methods can release welding residual stress to a certain extent, they have low efficiency and high cost. Presently, the new technique of laser welding with filling materials can reduce the defects of welding technology, increase the use rate of laser, and improve the performances of the welded joint, so domestic and foreign scholars are racing to study it [10,11,12]. Welding filling materials include wire and powder, and the latter is more frequently used as it is flexible and economical. 

The processability of laser welding with powder filler is similar with the laser-based additive manufacturing process, especially with selective laser melting. Selective laser melting (SLM), due to its capacity to fabricate complex freedom geometries directly from a computer-aided design (CAD) model, has been hailed as one of the most promising manufacturing technologies for net shape industrial scale production. For example, under the appropriate process conditions, the Al-Mg-Sc alloy (Scalmalloy) has been developed as a high-strength aluminum alloy for selective laser melting [13]. The tensile and fatigue strengths of the Scalmalloy exceed those of AlSi10Mg by 70%. Qiu C. L. et al. have prepared Ti-6Al-4V samples by selective laser melting, and have also studied the effect of laser scanning speed and powder layer thickness on the surface structure and porosity of Ti-6Al-4V samples [14]. In addition, the characteristics of Ti-6Al-4V alloy were correlated with the melt flow behavior through both experimental and modeling approaches. Thus, these research results provide the theoretical basis for the formation of welding seam with high performances in the process of laser welding with powder filling possesses. 

Because the Fe-Mn-Si SMAs has stress-accommodation characteristics [15,16], it can induce γ→ε martensitic and reverse transformation when the alloys is subjected to external stress, and this phase transformation can relax the external macro-stress and macro-deformation. For the above reasons, this work presents the technology for in situ formatting of the Fe-Mn-Si SMAs welding seam by laser welding with filler powder, aiming to use the stress-induced γ→ε martensite and reverse phase transformation, reducing welding residual stress and cracking and improving the performances of the weldment in the laser welding process and working process.

To prepare the Fe-Mn-Si SMAs welding seam under the optimum process in this paper, the methods were used as follows: (1) Single factor test and orthogonal test were used to optimize the welding conditions; (2) Restrained mixing uniform design method [5] was employed to form Fe-Mn-Si SMAs welding seam in the process of laser welding with powder filler; (3) Spectrophotometry method and the energy disperse spectroscopy (EDS) of scanning electron microscopy (SEM) were adopted to measure the mass fraction of Fe-Mn-Si SMAs welding seam in micro and macro. The distribution law of residual stress inside the laser welding specimens was studied by the combination of hole-drilling method and ANSYS finite element software. With the cyclic bending fatigue method and SEM, the bending fatigue life and the fracture morphology of weldments with Fe-Mn-Si SMA welding seam (test materials) and 304 stainless steel welding seam (comparative materials) were compared and analyzed. By the simplified one-dimensional thermodynamic model in the laser welding process, the strengthening mechanism of the Fe-Mn-Si SMAs welding seam was qualitatively studied. Additionally, by the crystal theory of stress-induced γ→ε martensite phase transition and the empirical formula of phase transition driving force in Fe-Mn-Si SMAs, the phenomenon whereby the residual stress in the Fe-Mn-Si SMAs welding seam is released as the drive force of phase transition was also explained.

## 2. Materials and Methods

### 2.1. Experiment of Laser Welding with Powder Filler

**Figure 1:**
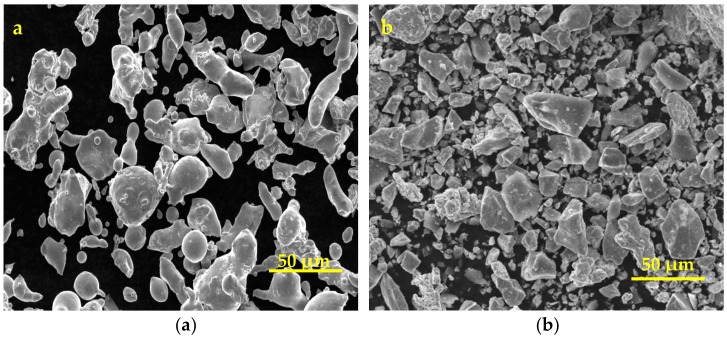
SEM pattern of (**a**) the 304 stainless steel powder and (**b**) the Fe/Mn/Si mixed powder.

The welding base metal was 304 stainless steel plate with dimensions of 100 × 50 × 2 (mm) and a groove size of 1 mm × 45° by EDM cutting—the angle between the workpieces is prepared for filling with powder. In addition, the aim of the groove is to reduce the effect of the substrate metal on the chemical component of the welding seam. The usual filling powder is the material which possesses the most similar mass fraction to the welding substrate, so 304 stainless steel powder was selected as a comparative material. To form the Fe-Mn-Si SMAs welding seam in situ, a mixed powder of Fe, Mn, Si, Cr, Ni elements was chosen as experimental material. The chemical compositions of the substrate metal, the contrast filling powder (304 stainless steel powder) and the experimental filling powder (Fe/Mn/Si mixed powder) are shown in Table 1. The particle size of the powder ranged from 50 μm to 100 μm, mixed by a QM-1 horizontal bowl mill for 4 h, and the SEM patterns of 304 stainless steel and Fe/Mn/Si mixed powder are shown in Figure 1.

**Figure 2:**
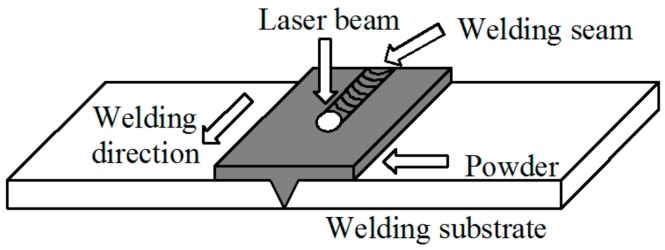
Schematic illustration of laser welding.

Prior to the experiment, acetone was used to clear the attachments on the surface of the specimen, and the powder, with thickness of 1 mm, was symmetrically pre-set and compacted at the butt-welding center of the 304 stainless steel by a self-made scraper. To avoid powder splashing by the shielding gas, shielding gas wasn’t used. A DL-LPM-V CO2 laser processing system with a maximum output power of 5 KW and a copper focusing lens with a focal length of 300 mm were selected. Based on the tensile strength, the optimum process parameters were obtained as follows: laser power of 2.7 KW, welding speed of 160 mm/min and defocusing amount of +5 mm. The schematic illustration of the laser welding is shown in Figure 2.

**Figure 3:**
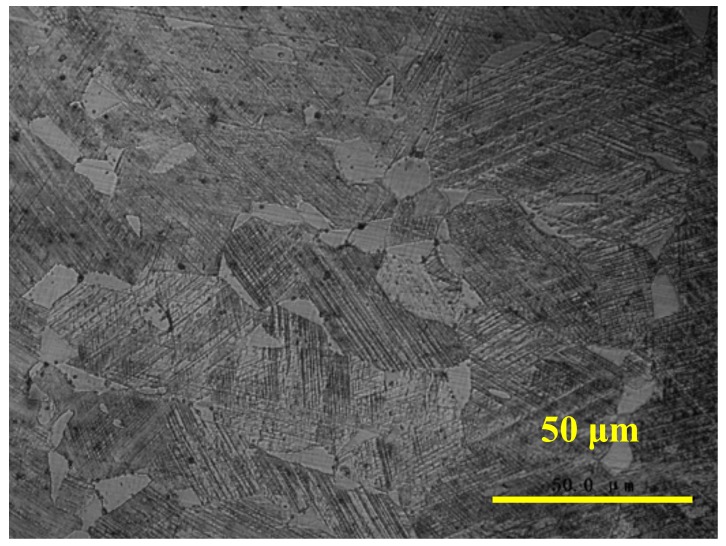
Microstructure of Fe-Mn-Si SMAs welding seam.

With the restrained mixing uniform design method [17], when the Fe/Mn/Si/Cr/Ni mixed powder mixture ratio is 21.118:57.207:21.675:0:0, the stainless Fe-Mn-Si (Fe16Mn5.5Si12Cr6Ni) memory alloy should be formed. Under the above process conditions, the base metal was laser welded with the specific Fe/Mn/Si mixed powder. By the SEM-EDS, the mass fractions of the welding seam were Mn 14.98, Si 5.50, Cr 11.54 and Ni 5.78. In addition, the components were also measured by the photometric analyzer, the result was Mn 15.234, Si 5.358, Cr 12.066 and Ni 6.097. Thus, the experimental results show that Fe15Mn5Si12Cr6Ni SMAs welding seam can be formed in situ by laser welding with powder filler at both micro and macro scales. To measure the shape recovery ratio of the Fe-Mn-Si SMA welding seam, a slender with dimensions of 100 mm × 2 mm × 0.5 mm was cut by an EDM cutting machine. After cleaning and solution treatment, the shape recovery rate of the alloy weld was 39.5%, as measured by the bending recovery test. Meanwhile, the recovery rates of the 304 stainless steel welding seam and the base metal were approximately 0% under the same treatment method. Meanwhile, the Fe-Mn-Si SMAs welding seam was operated with a solution treatment and 6% pre-deformation, and the microstructure of the Fe-Mn-Si SMAs welding seam was observed by the optical microscope after corrosion with CuSO_4_ solution, as shown in Figure 3. It can be seen from Figure 3 that the structure of the welding seam after pre-deformation is martensite, which indicates the stress inside the Fe-Mn-Si SMAs welding seam can induce the martensite phase transformation. Thus, the Fe-Mn-Si SMA welding seam not only possesses the mass fraction within the stainless Fe-based memory alloy range, but also has a shape memory effect and stress-induced martensite phase transformation characteristics.

**Figure 4:**
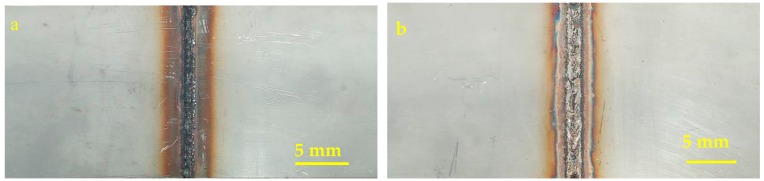
Surface morphology of (**a**) 304 stainless steel welding seaming and (**b**) Fe-Mn-Si SMAs welding seam.

**Figure 5:**
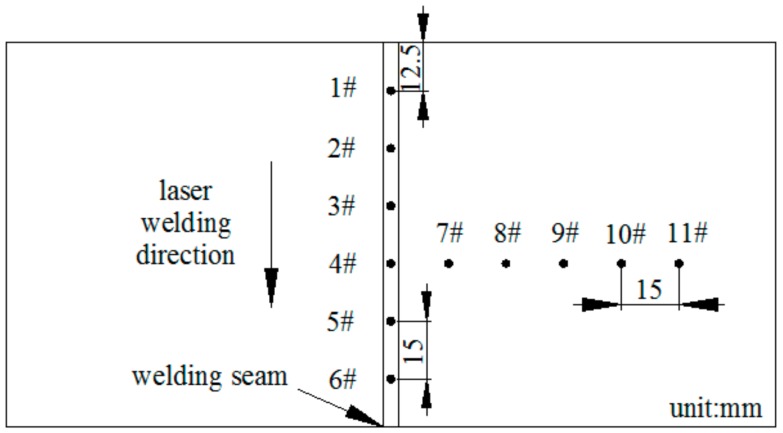
Layout schemes of residual stress measuring points.

This experiment comparatively studied the mechanical properties of the weldments with Fe-Mn-Si SMAs welding seam and 304 stainless steel welding seam; their appearances after laser welding are shown in Figure 4. The hole-drilling method was used to measure the residual stress, and the measuring positions are shown in Figure 5. The drilling equipment is HT-II magnetic drilling equipment, the diameter and depth of the hole are both 1.5 mm. The strain data was collected by the NI 9235 strain module and brought into the calculation formula to obtain the residual principal stress.

**Figure 6:**
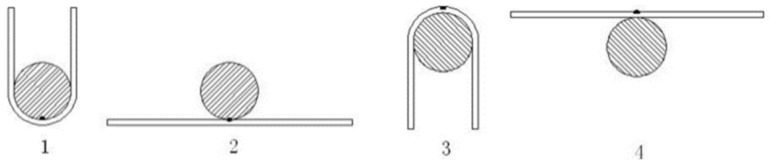
Schematic diagram of full cycle bending fatigue test.

Using the cyclic bending fatigue method, the bending fatigue life of the welded specimens was obtained, and the schematic diagram of the bending fatigue test is shown in Figure 6. The size of bending specimen and bending die were 100 mm × 4 mm × 2 mm and ф30 mm, respectively. The fracture morphology was observed by SEM.

### 2.2. Numerical Simulation of Laser Welding with Powder Filler

**Figure 7:**
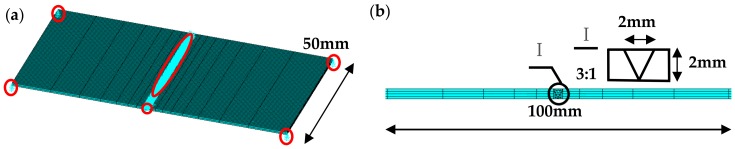
Displacement constraint (**a**) and mesh generation (**b**) of the finite element model.

The welding parameters, such as model size, laser energy, welding speed and defocusing amount, were consistent with actual machining conditions, and the finite element model is shown in Figure 7a. To ensure the accuracy and the efficiency of the numerical simulation, the SOLID90 element and SOLID70 element were selected as the simulated elements of the weld seam and the substrate, respectively. As Figure 7b shows, the mesh size of the welding seam is 0.5 mm, and it gradually increases as the simulated unit away from the cladding layer. 

The settings for the numerical simulation were as follows:

(1) Type of heat source: The energy distribution in the middle of the spot is more than that of the edges in the laser processing, which is similar to the Gaussian mathematical model. Thus, the Gaussian heat source model was used as the heat source, and the “birth and death unit” technology was adopted to simulate the metallurgical bonding between the filler powder and the substrate in the movement process of the laser spot [18].
(2) Material parameters: The thermo-mechanical indirect coupling method was adopted to simulate the laser welding stress field. That is, the temperature unit was converted into the stress unit when the simulation of the temperature field was completed, and the node temperature of each load step (time point) in the thermal analysis was regarded as a load of the stress field. The necessary material parameters for the simulation include: thermal conductivity (W/m·°C), density (kg/m^3^), specific heat capacity (J/kg·°C), enthalpy (J/m^3^), Poisson’s ratio, coefficient of thermal expansion (μm /m·K), elastic Modulus (Pa), yield Stress (Pa), etc. The material properties of 304 stainless steel and Fe-Mn-Si memory alloys were obtained by related literatures [19,20] and JMatpro software.
(3) Boundary conditions of temperature field: The boundary conditions of temperature field included radiation, convection and heat conduction. Because the size of the welding seam is small, the heat loss caused by the radiation can be ignored. The convective heat transfer coefficient was applied on the model surface to simulate the convection among the welding seam, the substrate and the surrounding media, and the temperature-dependent convection coefficient is achieved by the TABLE mode in the ANSYS software [21]. The heat conductivity coefficient was defined to simulate the heat conduction between the welding seam and the substrate.
(4) Boundary conditions of stress field: The boundary conditions of stress field were the constraints on the degree freedoms in the model (displacements in three directions). To avoid stress concentration and simulate a situation where the weldment was freely placed on the workbench, the displacement constraints were set as follows: the nodes in the refined unit of the welding seam were applied as a restraint in the vertical direction to the welding seam; the node in the initial position on the bottom of the welding seam was applied a restraint in the horizontal direction of the welding seam; 8 vertices in the substrate furthest away from the welding seam were set as the constraint in the vertical direction of the substrate surface. The schematic diagram of the displacement constraint is shown in Figure 7a.

## 3. Results and Discussions

### 3.1. Residual Stress Distribution of Welding Specimens

**Figure 8:**
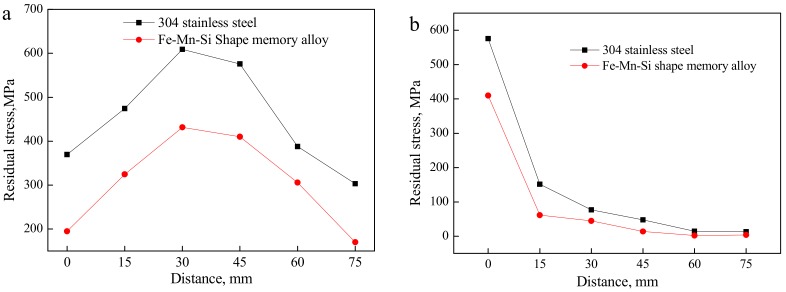
Distribution of residual stress inside laser welding specimen in directions (**a**) parallel and (**b**) perpendicular to the welding seam.

The strain values at each measurement point of the weldments with the 304 stainless steel welding seam and the Fe-Mn-Si SMAs welding seam were measured by the hole drilling method, and the measured values and the calculated residual stress are shown in Table 2 and Table 3. Meanwhile, the distribution curves of the residual principal stress *σ* in the direction parallel and perpendicular to the welding seam are shown in Figure 8.

**Figure 9:**
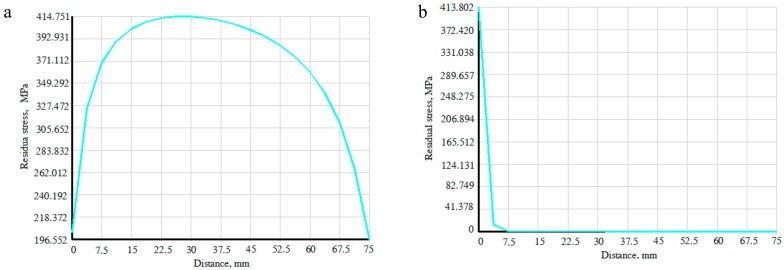
Distribution of residual stress inside the laser welding model in the directions (**a**) parallel and (**b**) perpendicular to the welding seam.

It can be seen from Figure 9 that the residual stress distribution laws at the measurement points of the Fe-Mn-Si SMAs welding seam and the 304 stainless steel welding seam are similar in the direction parallel or perpendicular to the welding seam. That is, along the direction of the welding seam, the welding residual stress is tensile stress and gradually decreases from the middle region toward the two sides of the welding seam. In the vertical direction of the welding seam, the residual stress is inversely proportional to the distance between the welding seam and measuring points, and it tends to a zero value [22].

The in situ formation of Fe-Mn-Si SMAs in the laser welding process was simulated under the same technological conditions, and the distribution of the residual stress in the direction of principal and perpendicular to the welding seam is shown in Figure 9a,b. It can be seen from Figure 9 and Figure 5 that the stress distribution law obtained by simulation and experiment possesses consistent, and the test results and simulation results mutually verify each other’s accuracies. However, some errors still exist, and there are two reasons for this. One reason is that the Gaussian heat source was an ideal heat source, while the surface state of the materials and the distribution of materials elemental components will lead to an uneven energy distribution in an actual laser processing heat source. The other is that the mesh size cannot match distribution size.

**Figure 10:**
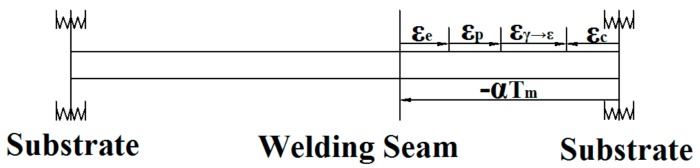
One-dimensional simplified model of laser weldment.

To analyze the distributional characteristics of the residual stress and the low residual stress phenomenon of the experimental material, it is necessary to understand the generation mechanism of the residual stress first. The welding seam is a gap before the powder melting and filling it during the laser heating process, so the welding seam only bears the tensile stress of the surrounding base metal in the process of cooling from high temperature. The restrained end of the welding seam changes from a molten-state metal to the solid-state parent metal, so the complicated constraint type is assumed to be elastic constraint, and the contraction strain of the welding seam is represented by *ε_c_* when it cools completely. After the weldment cools to room temperature, the deformation of the welding seam is composed of the following five parts: the thermal strain *ε_T_*, the elastic strain *ε_e_*, the plastic strain *ε_p_*, the transformation strain *ε_γ_*→*_ε_* and the contraction strain *εc*. Thus, to analyze the stress and deformation of the welding seam in the cooling process from the high temperature T_m_ to the room temperature 20°C, the weldment was simplified in a one-dimensional model [23], as shown in Figure 10.

In the simplified model of laser weldment, the slender expresses Fe-Mn-Si SMA welding seam, and the two ends are base metals. In addition, the strain variation in the weldment during the laser welding is shown in Equation (1).

Δε = ε_T_ + ε_e_ + ε_p_ + ε_γ→__ε_ = −ε_c_ (1)

where, the thermal strain *ε_T_* and the contraction strain *ε_c_* are caused by temperature reduction and the elastic contraction of base metal, respectively, and both are compressive strain. In addition, the elastic strain *ε_e_*, the plastic strain *ε_p_* and the phase transformation strain *ε_γ_*_→_*_ε_* are caused by the thermal strain, and the sum between the absolute value of *ε_e_*, *ε_p_* and *ε_γ_*_→_*_ε_* is equals to the difference between the absolute of *ε_T_* and *ε_c_*. Thus, the strain directions of *ε_e_*, *ε_p_* and *ε_γ_*_→_*_ε_* are opposite to that of *ε_c_*, former transition strains are both tensile strain. In this test, the plastic strain that exceeds the elastic strain (yield limit) at room temperature is the residual strain, as shown in Equation (2).

εp = − ε_T_ − ε_e_ − ε_γ__→ε_ − ε_c_ (2)

With the law of linear expansion, the thermal strain is shown in Equation (3).

*ε_T_ = α* × (20 − *T_m_*) (3)

where, *α* is the average coefficient of linear expansion of the alloy, and (20 − *T_m_*) is the variable quantity of the temperature in the cooling process of laser welding. According to Hooke’s law, the welding residual stress is shown in Equation (4).

*σ_P_*= E[*α* × (*T_m_*− 20) − *εe* − *ε_γ_*_→_*_ε_* − *ε_c_*] (4)

For the same materials (test materials or comparative materials), its phase transformation strain and temperature variation are basically the same in their respective measurement points. Along the direction of the welding seam, the linear expansion coefficient, the elastic limit and phase transition strain of the materials in each measurement point are relatively similar, but the constraint degree in the middle region of the welding seam is more than that in the two sides. Thus, the former contraction strain *εc* is smaller, and the residual stress is larger in the middle region, and gradually reduces toward the two sides. In the vertical direction to the welding seam, the temperature variation (thermal strain *ε_T_*) of the measuring point gradually decreases as it far away from the center of the welding seam, so the welding residual stress gradually decreases.

**Figure 11:**
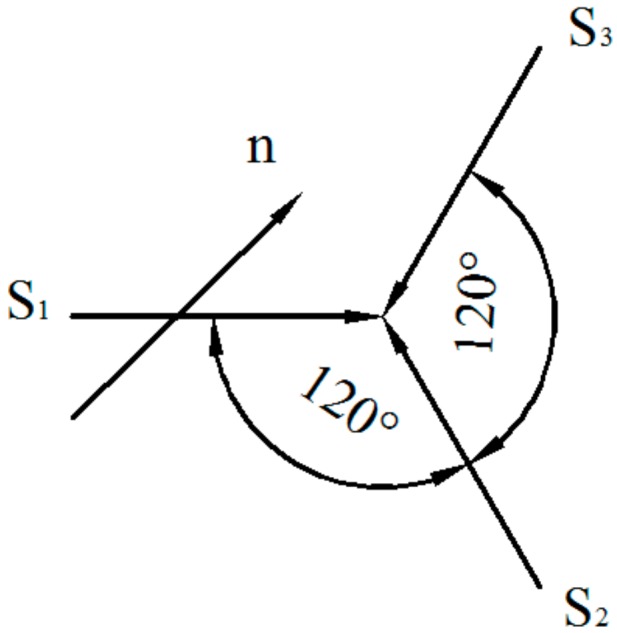
Shear strains caused by equivalent ε variants in one habit plane.

Although the distribution law of residual stress inside the test materials and the comparative materials are the same, the former residual strain is lower due to its stress-induced martensitic transformation strain *ε_γ_*_→_*_ε_*. The mechanism of the residual stress reduction inside the test materials is shown as follows: under the constrained state of solidified base materials, the residual stress is regarded as a phase transition deformation force to drive crystal orientation a/6 <112>_γ_ sweeping every other crystallographic plane families {111}_γ_ in the direction of crystallographic orientation families <112>γ when the residual stress acts on the γ-austenite of the welding seam in the temperature range from the Ms (martensitic transformation starting temperature) to the Md (stress-induced martensitic transformation ending temperature), and the ε-martensite is formed by the single-oriented Shockley imperfect dislocation movement. During the martensitic transformation, the formed ε-martensite can generate three equivalent a/6<112> shear strains **S_1_**, **S_2_**, and **S_3_** (with a mutual spacing of 120°) in a {111}_γ_ habit plane, and the welding residual stress in this test materials will cause the ε-martensite, which produces a macro shearing strain along the **n** direction (residual stress direction). Supposing the angle of the **n** direction with the {111}_γ_ plane and the **S_1_** are *θ* and *α* (Figure 11), respectively, then Equations of the macro shearing strain caused by equivalent ε variants in one habit plane are shown as Equation (5).

**S_1n_**= **n**·**S_1_** = p_1_**S_0_**cos*θ*cos*α* (5)

**S_2n_**= **n**·**S_2_**= p_2_**S_0_**cos*θ*cos(120 + *α*) (5)

**S_3n_**= **n**·**S**_3_ = p_3_**S_0_**cos*θ*cos(120 − *α*) (5)

where, **S_0_** is the maximum strain caused by a single variant, and its value is equal to 8^−1/2^ (35.36%). **S_1n_**, **S_2n_** and **S_3n_** are the macroscopic strains caused by the shear strains **S_1_**, **S_2_** and **S_3_** in the **n** direction, respectively, and the p_1_, p_2_, p_3_ is the generation probabilities of equivalent shearing strains **S_1_**, **S_2_** and **S_3_** caused by residual stress in the habit plane. Thus, the total macroscopic strains along the **n** direction is shown as follows: **S** = **S_1n_** + **S_2n_** + **S_3n_** = **S_0_**·cos*θ*·[p_1_·cos*α* + p_2_·cos(120 + *α*) + p_3_·cos(120 − *α*)] [24]. Because the single ε variant is always formed in the process of stress-induced ε-martensitic phase transformation under most conditions, for instance, p_1_ = 1, p_2_ = p_3_ = 0, then **S** = **S_0_**cos*θ*cos*α*. Meanwhile, the maximum macro shear strain **S** = **S_0_** = 35.36% can be obtained along the direction of **S_1_** shearing strain when *θ* = 0 and *α* = 0. Although the practical condition is different from the ideal condition, and the former macroscopic shearing strain is decreasing in the stress-induced γ→ε martensitic phase transformation, a certain macroscopic strain (0 < S < 8^−1/2^) is also present in the experimental materials. In addition, the possible practical conditions are shown as follows: (1) the martensitic phase transition possesses partial cooperation, that is, the phenomenon p_1_ ≠ p_2_ ≠ p_3_, and *θ* ≠ 0, *α* ≠ 0; (2) the γ→ε phase transitions are simultaneously performed in several {111}_γ_ habit planes to form multi-orientation ε-martensite; (3) the orientation of crystal grains in the polycrystalline alloy is different. Through the above analysis, the stress-induced martensitic transformation will cause tensile plastic strain in the direction of the residual stress *ε_p_*. This tensile plastic strain will resist the residual thermal contraction strain, that is, the phase transition deformation can relax the residual strain [25].

Furthermore, from the aspect of the phase transition free energy in the Fe15Mn5Si12Cr6Ni SMAs, the work in the stress-induced martensite phase transformation (ΔG) is equivalent to the free energy variation in driving the phase transformation. That is, the driving force for the martensitic phase transition can be provided by the external or internal stresses. Therein, the strain in the phase transition process includes two parts: the shearing strain along the habit plane, and the expansion strain perpendicular to the habit plane. Thus, the mechanical driving energy in the aspect of the martensite phase transformation strain can also be expressed in Equation (6) [26].

ΔG = 0.5*σ_S_*[(*s*·sin2*θ*·cos*α* ± *ξ*(1 + cos2*θ*)] (6)

where, *σ_S_* is the stress, *ξ* is the expansion strain perpendicular to the habit plane, *θ* is the angle between the stress and the habit plane, and *α* is the angle between the shearing direction and the maximum shear stress, the signs “+” and “−” express tensile stress and compressive stress, respectively.

Thus, the martensitic sheet is first formed along the direction of the maximum ΔG when the residual stress induces the martensitic phase transformation in the multicrystal, and the residual stress is released as a driving force during the lattice generating the shearing strain.

### 3.2. Bending Fatigue Life of Welding Specimens

**Figure 12:**
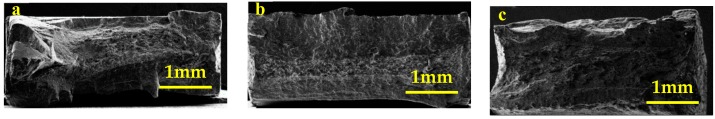
Macroscopic fracture surface of (**a**) 304 stainless steel substrate, (**b**) experimental materials and (**c**) comparative materials.

Through the bending fatigue test, the 304 stainless steel base metal, the weldment with Fe-Mn-Si SMAs welding seam and the weldment with 304 stainless steel welding seam possess 274, 249 and 136 bending fatigue cycles, respectively, and their macroscopic fracture morphology is shown in Figure 12. The fatigue cycles of the experimental materials and comparative materials are 49.6% and 90.9% of the base metal, respectively, and it can be seen from Figure 8 that the fatigue fracture consists of a crack source zone, crack propagation zone and fatigue final rupture zone.

**Figure 13:**
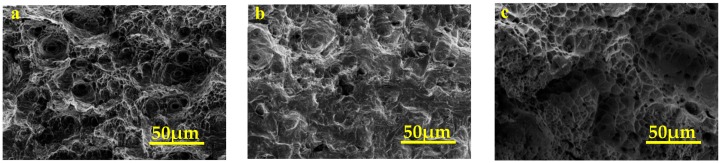
Fatigue final rupture zone surface of (**a**) 304 stainless steel substrate, (**b**) experimental materials and (**c**) comparative materials.

Figure 12 shows that the fatigue cracks originated on the surface of the specimen, and the cracks gradually grew and extruded to form crack propagation zones that are centered on the fatigue crack source under the alternating stress. Then the effective cross section is gradually induced, with the cracks extending, and the specimen immediately fracturing to generate a fatigue final rupture zone when the stress increases beyond the breaking limit. Because three specimens possess similar crack source zones (surface crack source) and crack propagation zones (fatigue striations and few tearing ridges), this won’t be discussed in detail. It can be seen from the macroscopic morphology and the fatigue final rupture zone of each fracture in the Figure 12 and Figure 13, and the results are shown below. The macro fracture of the 304 stainless steel base metal is prominent, and the fatigue final rupture zone is significantly higher than the crack source and the crack propagation zone. This is because the base metal has better bending fatigue performance, and the necking occurred in the local area between the metal interfaces and the inclusions in the fatigue final rupture zone. Then numerous microscopic holes are necking, growing, gathering and connecting, and the base metal is finally fracturing and forming uneven dimple-like when the section size of the necking area reduced to a certain extent. This fracture pattern belongs to plastic fracture, and reflects the fact that the base metal possesses better bending fatigue properties [27]. The macro fracture morphology of the experimental materials is slightly flatter than that of the base metal, and the former fatigue final rupture zone is also composed of dimples. However, the dimples’ depth and number in the experimental materials are less than those of the base metal, so the former bending fatigue characteristics are reduced. Meanwhile, the macro fracture of the weldment with 304 stainless steel welding seam is particularly smooth, and its fatigue final rupture zone consists of a large number of tearing ridges. This fracture belongs to a cleavage fracture (brittle fracture), and its bending fatigue performance is poor. 

Compared with the number of bending fracture cycles and fracture morphologies of the test materials and the comparative materials, the former bending fatigue performance is greatly improved. The reasons are as follows: (1) The residual stress inside the Fe-Mn-Si SMAs welding seam is induced martensitic phase transformation in the laser welding process, and the welding residual stress is released to decrease the stress concentration of the welding joint and improve the bending fatigue strength; (2) The Fe-Mn-Si SMAs possess stress self-accommodation characteristics. That is, an additional deformation can be generated by stress-induced γ→ε martensite forward and reverse transformation during the strain cycle, and this deformation in the experimental materials can absorb energy, suppress the plastic slip deformation and reduce the accumulation of dislocations to improve the strain fatigue properties.

### 3.3. Microhardness Distribution of Welding Specimens 

**Figure 14:**
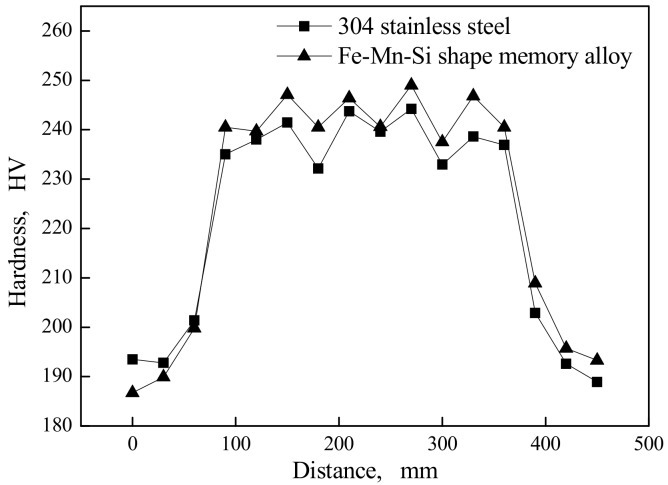
Microhardness distribution of welding specimens.

Under the same technological conditions, the microhardness distribution of the laser welded specimen with the Fe-Mn-Si SMAs welding seam and 304 stainless steel welding seam can be seen in Figure 14. As shown in Figure 14, from the substrate to the welding seam, the microhardness jumps to a high value. The reason is that the heating and cooling speed is rapid in the laser welding process, which leads to the grain size of the welding seam smaller than that of the substrate, and the strengthening effect of the tiny grain will enhance the microhardness. The average hardness of the experimental material and comparative material in the welded joint zone is HV 242.8 and HV 238.2, respectively. The microhardness of the former is slightly higher than that of the latter. On one hand, this is because the high microhardness martensite exists in the welding seam of experimental material. On the other hand, the great mass fraction of Mn, and Si elements in the welding seam of experimental material will benefit to refine the grain.

## 4. Conclusions

In this test, the optimal welding process conditions were obtained, and the Fe-Mn-Si SMAs welding seam was in situ formed under these conditions. Meanwhile, the mechanical properties of the experimental materials and comparative materials were researched, and the former strengthening mechanism was analyzed. Based on the above discussion, the following conclusions can be drawn:

1. Under the optimal technological conditions (the laser power is 2.7 KW, the scanning speed is 160 mm/min, the defocus amount is +5 mm) and the mass ratio of Fe/Mn/Si powder (21.118:57.207:21.675), the Fe15Mn5Si12Cr6Ni SMAs welding seam was formed in situ in laser welding of 304 stainless steel with the Fe/Mn/Si powder.
2. In the parallel direction to the laser welding, the residual stress of the measuring points in the middle of the welding seam is greater than that in the two sides. In the perpendicular direction to the welding seam, the peak value of the residual stress exists in the narrow area of the welding seam. As the distance between the measuring points and the welding seam increases, the stress rapidly decreases and gradually approaches zero MPa. Although the distribution law of residual stress in the test materials and the comparative materials is similar, the former residual stress at the corresponding measurement points is smaller. Furthermore, the results of the numerical simulation and the experiment are mutually consistent.
3. The weldment with Fe-Mn-Si SMAs welding seam and 304 stainless steel welding seam possess 249 and 136 bending fatigue cycles, respectively, and the fatigue cycle of the experimental materials and comparative materials is 90.9% and 49.6% of the base metal, respectively. The macro fracture morphology of the comparative materials is smoother than that of the test materials, and their appearance of fatigue final rupture zone are dense tearing ridges and large number of dimples in turn, which indicates that the Fe-Mn-Si SMAs welding seam significantly improved the bending fatigue characteristics of the weldments.
4. The experimental materials have slightly greater microhardness than the comparative material, and the microhardness jumps from the substrate to the welding seam.
5. The residual stress can induce γ→ε martensitic phase transformation in Fe-Mn-Si SMAs welding seam. In addition, the strengthening mechanism of experimental materials is shown as follows: (1) the deformation in the γ→ε martensitic phase transformation can resist residual compression strain, which relaxes the residual strain; (2) the residual stress, as the phase transformation driving force, can be released in the process of shear deformation.

## Figures and Tables

**Table 1:** Chemical compositions of 304 stainless steel, 304 stainless steel powder and Fe/Mn/Si mixed powder (wt. %).

Material	Chemical Composition (%)					
	Cr	Ni	Mn	Si	C	Fe
304 stainless steel	19.14	8.71	1.15	0.40	0.061	Bal.
304 stainless steel powder	18.75	10.45	1.54	0.93	0.039	Bal.
Fe/Mn/Si mixed powder	-	-	57.20	21.68	-	21.12

**Table 2:** Strain value *ε*_1_, *ε*_2_, *ε*_3_ and stress value *σ* of measuring points inside the specimen with 304 stainless steel welding seam.

	Strain and Stress	*ε*_1_/10^−6^	*ε*_2_/10^−6^	*ε*_2_/10^−6^	*σ/*MPa
Measuring Points					
1#		161.666	−132.227	−60.987	369.657
2#		75.849	−115.549	−378.550	474.321
3#		78.161	−101.818	−477.033	608.969
4#		70.736	−75.033	−440.693	575.674
5#		100.995	−71.499	−285.726	387.764
6#		102.961	−131.863	−109.561	303.173
7#		−58.234	59.018	54.177	151.497
8#		−15.813	39.283	81.449	76.872
9#		21.333	71.828	87.594	47.624
10#		21.303	18.095	35.175	14.542
11#		7.542	6.307	20.541	13.117

**Table 3:** Strain value *ε*_1_,* ε*_2_,* ε*_3_ and stress value *σ* of measuring points inside the specimen with Fe-Mn-Si SMAs welding seam.

	Strain and Stress	*ε*_1_/10^−6^	*ε*_2_/10^−6^	*ε*_2_/10^−6^	*σ/*MPa
Measuring Points					
1#		23.919	−97.578	−152.341	195.148
2#		15.107	−155.331	−283.961	324.452
3#		26.326	−206.429	−391.872	431.596
4#		−51.699	−132.102	−365.205	410.049
5#		−100.039	−96.584	−279.449	305.745
6#		60.581	−26.194	−115.464	170.159
7#		−16.241	54.402	−69.058	61.440
8#		−47.992	132.402	−133.994	44.797
9#		76.052	49.032	65.042	13.977
10#		28.053	23.773	33.498	2.162
11#		14.556	21.381	18.224	3.542
